# Treatment of an Erratic Extraction Socket for Implant Therapy in a Patient with Chronic Periodontitis

**DOI:** 10.1155/2016/1746961

**Published:** 2016-10-11

**Authors:** Yusuke Hamada, Srividya Prabhu, Vanchit John

**Affiliations:** Department of Periodontics and Allied Dental Program, Indiana University School of Dentistry, Indianapolis, IN 46204, USA

## Abstract

As implant therapy becomes more commonplace in daily practice, preservation and preparation of edentulous sites are key. Many times, however, implant therapy may not be considered at the time of tooth extraction and additional measures are not taken to conserve the edentulous site. While the healing process in extraction sockets has been well investigated and bone fill can be expected, there are cases where even when clinicians perform thorough debridement of the sockets, connective tissue infiltration into the socket can occur. This phenomenon, known as “erratic healing,” may be associated with factors that lead to peri-implant disease and should be appropriately managed and treated prior to surgical implant placement. This case report describes the successful management of an erratic healing extraction socket in a 62-year-old Caucasian male patient with chronic periodontitis and the outcomes of an evidence-based treatment protocol performed prior to implant therapy. Careful preoperative analysis and cone beam computed tomography imaging can help detect signs of impaired healing in future implant sites and prevent surgical complications.

## 1. Introduction

Over the past decade, the oral rehabilitation of fully or partially edentulous patients with dental implants has become routine in everyday clinical practice. Implants are placed either in sockets following the extraction of teeth, in sockets following grafting and healing, or in fully healed native bone. The characteristics and progression of healing in extraction sockets have been extensively investigated in animal models and in human clinical trials [1–3]. These studies have included clinical and radiographic dimensional changes, as well as histological analyses [4]. A systematic review of the existing literature assessed the magnitude of dimensional changes for hard and soft tissues of the alveolar ridge up to 12 months after tooth extraction in humans. The results from human reentry studies show that 29–63% horizontal bone loss and 11–22% vertical bone loss occur 6 months following tooth extraction [5]. Current evidence indicates that alveolar ridge preservation treatment at the time of extraction can minimize the degree of ridge dimension shrinkage [6, 7]. Not all patients consider or plan on future implant therapy at the time of their extractions, so they may go without the alveolar ridge preservation procedure. This ultimately can have an effect on ridge dimensional change and the healing process following extraction.

The sequence of cellular and tissue healing following tooth extraction in humans starts with blood clot formation within the socket; the clot is then replaced by granulation tissue, and, subsequently, osteoid formation occurs [1]. Histologic evaluation of extraction sites has primarily been investigated with teeth devoid of pathological features. In daily practice, many extracted teeth are periodontally or endodontically involved or are extracted from medically compromised individuals. In some cases, even when surgeons perform thorough debridement of the sockets, connective tissue infiltration into the socket can occur [8]. Some reports showed that bacterial contamination during implant insertion and premature loading, bone microfractures, and the presence of a preexisting inflammation (bacteria, inflammatory cells, and/or remaining cells from a cyst or granuloma) are the etiologic factors of retrograde peri-implantitis. Retrograde peri-implantitis is often accompanied by symptoms of pain, tenderness, swelling, and/or the presence of a sinus tract. The appropriate treatment methods for retrograde peri-implantitis are still unclear [9, 10]. Kim et al. defined “erratic healing” as healing where fibrous scar tissue is found occupying the extraction site rather than bone after 12 or more weeks of healing. Erratic healing is a not a rare complication. In their retrospective study, the authors showed that, in 5.71% of subjects receiving extractions, 4.24% of extraction sites demonstrated some degree of erratic healing sites [11]. Appropriate treatment for extraction sites with erratic healing is needed prior to or at the same time of implant surgical therapy to maintain long-term stability of the implant. The purpose of this paper is to illustrate a clinical case and suggest an evidence-based treatment protocol for sockets with erratic healing prior to implant placement.

## 2. Case Presentation

**Figure 1:**
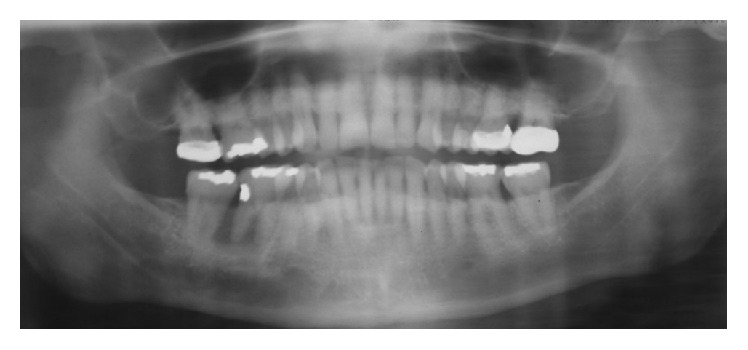
Panoramic radiograph at the initial appointment (#30 was extracted by the referring dentist).

**Figure 2:**
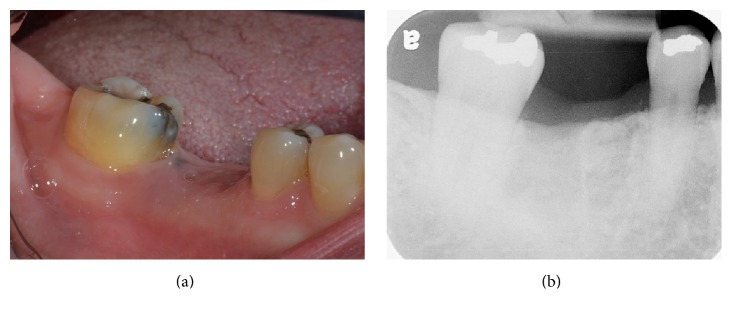
Preoperative views: (a) intraoral; (b) periapical radiograph.

A 62-year-old Caucasian male was referred to the Graduate Periodontics Clinic at Indiana University School of Dentistry, Indianapolis, Indiana, USA, from a general dentist's office for periodontal treatment. The patient reported a history of hypertension, coronary artery blockage with stent replacement in 2006, osteoarthritis, and hyperlipidemia. The clinical examination demonstrated increased periodontal probing depths, up to 6 mm, on the posterior teeth. Tooth #30 had previously been extracted due to a combained periodontic-endodontic lesion (Figure 1). The radiographic examination revealed horizontal bone loss on the posterior teeth. The periodontal diagnosis was generalized mild chronic periodontitis with localized moderate chronic periodontitis associated with teeth #2, 3, 14, 15, 18, and 19 [12]. The patient's oral hygiene was considered to be acceptable. An O'Leary plaque score of 29% was recorded at the initial appointment. The possible treatment interventions for the periodontitis and edentulous ridge #30 were explained to the patient. These included (1) oral hygiene instructions, (2) nonsurgical periodontal therapy, (3) surgical intervention (resective osseous treatment) around posterior teeth, and, following good control of the patient's periodontal condition, (4) replacement of #30 with a dental implant-supported restoration. The goal of the anti-infective therapy (nonsurgical therapy) was to reduce the bacterial load and inflammation. The patient underwent a periodontal maintenance session and received individualized oral hygiene instructions. His oral hygiene improved, and an O'Leary plaque score of 9% was noted. Shortly after the periodontal maintenance appointment, osseous resective surgery on UL, LL, and UR quadrants was rendered to achieve shallower probing depth and a better periodontal environment for the posterior teeth prior to implant surgery on #30. The patient elected to follow through with implant therapy for #30 with a single implant-supported crown (Figure 2).

**Figure 3:**
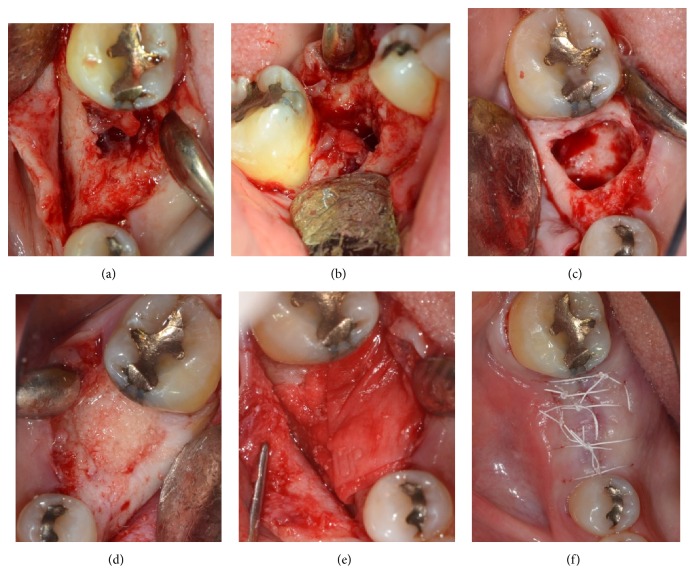
Guided bone regeneration procedure was performed on the erratic extraction site (a–f). After a full-thickness flap was elevated, granulation tissue was noted filling the previously extracted site (a and b). Following thorough debridement and irrigation, the site was determined to be a containable defect (c). FDBA was gently packed in the socket and covered with non-cross-linked collagen membrane (d and e). Complete primary closure was achieved with a combination of horizontal mattress and single interrupted sutures (f).

**Figure 4:**
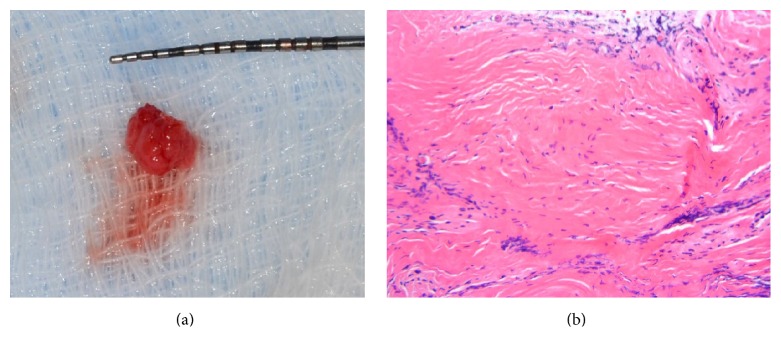
The removed specimen and histopathological picture (H&E stain). Approximate 5 × 5 × 3 mm elastic hard granulation tissue was removed from the erratic extraction site (a). Fibrous connective tissue with inflammatory cells infiltration (b).

On the day of implant placement on #30, infiltration of 2% lidocaine with 1 : 100,000 epinephrine was administered. Following a crestal incision over the edentulous ridge of #30 and intrasulcular incisions along the distal surface of #29 and mesial surface of #31, a full-thickness flap was reflected. After flap reflection, granulation tissue was noted filling the crestal area of the #30 extraction socket. The buccal and lingual walls of the socket were intact. Thorough debridement was attempted. Gaining access to the bottom of the defect was challenging due to the complexity of the defect shape. A small amount of crestal bone was removed with a high-speed handpiece and round burs to allow the surgeon access to the apex of the bony defect. Granulation tissue was removed from the socket and submitted for pathological examination to obtain a formal diagnosis. Following thorough irrigation with saline, hydrated freeze-dried bone allograft (FDBA: particle size 250 *μ*m–1000 *μ*m, Sunstar) was grafted into the socket and covered with Bio-Gide (non-cross-linked porcine collagen membrane, Geistlich). Primary closure of the site was obtained with single interrupted and horizontal mattress sutures, using 4-0 Cytoplast suture material (Figure 3). Postoperative instructions were given, and the patient was prescribed 500 mg amoxicillin, to be taken three times daily for 1 week. He was instructed to rinse for 30 seconds twice daily with 0.12% chlorhexidine gluconate for 2 weeks. Sutures were removed at 2 weeks after the surgical procedure. The site healed uneventfully, and the pathology report revealed that the tissue sample presented with edema along with intense lymphoplasmacytic infiltrate. The bulk of the specimen consisted of dense, hyalinized mineralized debris. The diagnosis given was residual chronically inflamed granulation tissue with fibrous connective scar tissue (Figure 4).

**Figure 5:**
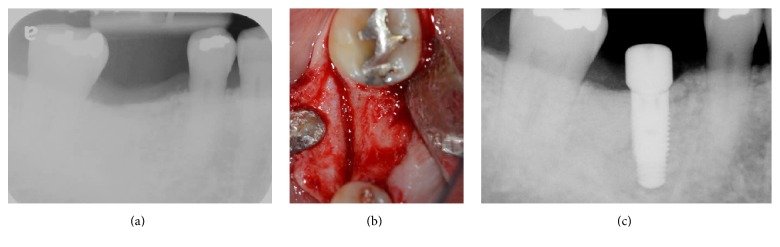
Implant placement five months after the graft procedure. The erratic healing extraction site showed increased radioopacity five months after the bone graft surgery (a). Bone fill was found on the crestal portion of the site (b). Implant was placed at a restoratively driven position (c).

**Figure 6:**
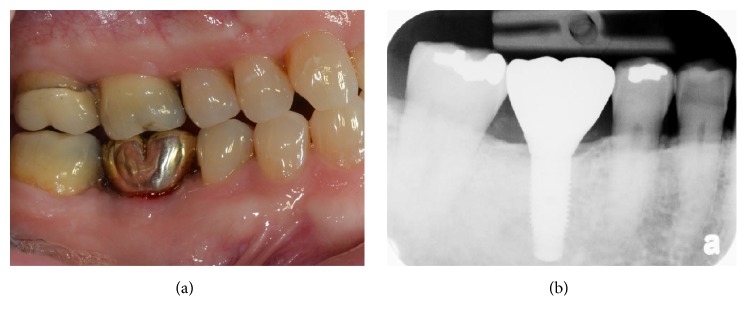
One-year follow-up after crown delivery. There is no evidence of inflammation or symptoms (a). Crestal bone loss or typical retrograde peri-implantitis was not noted on the periapical radiograph (b).

Five months after the graft procedure, implant placement was planned. Preoperative periapical radiograph showed increased bone density in the #30 site. Following a crestal incision and full-thickness flap elevation, a surgical guide was mounted intraorally, and an osteotomy was created as per the manufacturer's instructions. No soft tissue or granulation tissue was found within the osteotomy site. A Zimmer implant 4.7 × 11.5 (*D* ×* H*) was placed with insertion torque of 35 N/cm (Figure 5). An Osstell device was used to measure the stability, and an ISQ (Implant Stability Quotient) of 76 was measured from buccal and lingual positions. No adverse events were noted during the osteointegration phase. A custom abutment and a gold cast crown were delivered by a restorative dentist. Radiograph and clinical features showed soft and hard tissue stability at one year following crown delivery, without any symptoms or radiographic evidence of retrograde peri-implantitis (Figure 6).

## 3. Discussion

Observations of radiolucencies and the presence of fibrous scar tissue occupying an extraction socket rather than bone precluded the placement of a dental implant in that site. A retrospective study elucidated factors potentially impeding healing in post-extraction sites with computerized tomography. This study revealed that maxillary incisor/canine sites showed the lowest prevalence of erratic healing, whereas mandibular molar sites had the highest prevalence. The results of the multivariable analysis indicated that erratic healing was more likely to occur in subjects who are <60 years old (OR = 2.23), subjects with hypertension (OR = 2.37), molar sites (OR = 4.91), and single tooth extraction sites (OR = 2.98). This case report fits into all of these conditions except age [11].

In this case report, the buccal and lingual bone of the patient's extraction site was intact, and there was more than 2 mm of thickness on both sides. Additionally, the edentulous site was free of inflammation, which allowed the surgeon to obtain primary closure. Primary closure is one of the keys that allows for optimal results with guided bone regeneration. Wang and Boyapati [13] described those major biologic principles as “PASS” for predictable bone regeneration: “Primary wound closure” to ensure undisturbed and uninterrupted wound healing, “Angiogenesis” to provide necessary blood supply and undifferentiated mesenchymal cells, “Space maintenance” to facilitate adequate space for bone ingrowth, and “Stability of wound” to induce blood clot formation and an uneventful healing process. This case satisfied those four factors at the time of the graft procedure since the defect shape was containable and healthy soft tissue was available to cover the defect. However, almost 85% of erratic healing extraction sites presented with loss of either or both of the buccal and lingual walls [11]. If a defect is not containable, it is difficult to stabilize the wound, and a more rigid membrane or space maintainer, such as titanium-reinforced membrane or titanium mesh, may be needed to prevent tissue collapse [14]. Moreover, if a defect is noncontainable and large amount of bone augmentation is required to develop the implant site, placement of a bone graft material with a slow resorption rate (Deproteinized Bovine Bone Minerals, DBBM, etc.) and application of growth factors are suggested. Nevins et al. utilized recombinant human platelet-derived growth factor BB (rh-PDGF-BB) to regenerate large alveolar extraction sites with tenting screws, DBBM, and collagen membranes [15]. In this case series, eight sites were treated, and all sites healed successfully with evidence of bone-like hard tissue that was confirmed histologically as vital bone around the remaining graft particulate.

Since the erratic healing extraction site in this case report showed enough buccal-lingual ridge dimension to accommodate an implant fixture, the authors planned the implant surgery using cast models and periapical radiographs. The surgical plan changed unexpectedly during the first surgery. The clinical discovery of granulation and scar tissue in extraction socket was explained to the patient during the surgery, and the surgical plan was changed. However, a more careful presurgical examination, including cone beam computed tomography (CBCT), should be performed to minimize the risk of altering the surgical plan. The detection of erratic healing extraction lesions prior to surgery is beneficial for both patients and surgeons in order to discuss and create a comprehensive treatment plan that includes the duration and cost of the treatment needed. The American Academy of Oral and Maxillofacial Radiology (AAOMR) published a position statement regarding CBCT for dental implantology. The AAOMR recommends that cross-sectional imaging be used for the assessment of all dental implant sites and that CBCT be the imaging method of choice for gaining this information [16]. The decision to perform a CBCT examination must be clinically justified and conducted based on professional judgment (i.e., the judgment of the clinician that a CBCT image will potentially provide information needed for prosthetic treatment planning, implant selection, and/or surgical placement).

## 4. Conclusions and Practical Implication

This case report demonstrates successful treatment of erratic healing of an extraction site with guided bone regeneration. This therapy resulted in oral rehabilitation of edentulous site with a dental implant-supported restoration without any complications for one year after the prosthesis was delivered. Impaired extraction sites can be treated adequately if basic principles of biology, such as “PASS” principles, are followed. However, additional radiographic analysis with CBCT to detect a lesion prior to opening a surgical flap is highly useful for diagnosis and treatment planning.
